# COVID-19 pandemic and enrollment of critically Ill children in randomized clinical trials

**DOI:** 10.3389/fped.2025.1704390

**Published:** 2025-11-25

**Authors:** Sarah B. Kandil, David Panisello-Manterola, Madhuradhar Chegondi, Christine Allen, Jill M. Cholette, Michele Kong, Matthew Pinto, Hilary Schreiber, Christie Glau, E. Vincent S. Faustino

**Affiliations:** 1 Department of Pediatrics, Section of Critical Care Medicine, Yale University, School of Medicine, New Haven, CT, United States; 2 Lake Erie College of Osteopathic Medicine, Elmira, NY, United States; 3 Division of Pediatric Critical Care Medicine, University of Illinois College of Medicine, and Children’s Hospital of Illinois at OSF HealthCare, Peoria, IL, United States; 4 Division of Pediatric Critical Care, Department of Pediatrics, University of Oklahoma Health Sciences Center, Oklahoma, OK, United States; 5 Division of Pediatric Critical Care Medicine, Department of Pediatrics, University of Rochester Golisano Children’s Hospital, Rochester, NY, United States; 6 Department of Pediatrics, University of Alabama at Birmingham, Birmingham, AL, United States; 7 Division of Pediatric Critical Care Medicine, Department of Pediatrics, Maria Fareri Children’s Hospital at Westchester Medical Center, Valhalla, NY, United States; 8 Department of Pediatrics, Medical College of Wisconsin, Milwaukee, WI, United States; 9 Department of Anesthesiology and Critical Care Medicine, University of Pennsylvania Perelman School of Medicine and Children’s Hospital of Philadelphia, Philadelphia, PA, United States

**Keywords:** pediatrics, parental consent, patient selection, thromboprophylaxis, pandemic

## Abstract

**Objective:**

To evaluate the association of the COVID-19 pandemic with enrollment rates of critically ill children in randomized clinical trials (RCT). We hypothesized that enrollment rates declined due to increased parental refusal.

**Design:**

Cross-sectional analysis of 2 multicenter RCTs conducted pre- and post-COVID-19.

**Setting:**

A total of 5 centers pre-COVID-19 and 15 centers post-COVID-19 conducting pediatric RCTs on enoxaparin prophylaxis against catheter-associated thrombosis.

**Patients:**

Critically ill children <18 years old with newly inserted central venous catheters.

**Interventions:**

Randomization to enoxaparin prophylaxis or usual care.

**Measurements:**

Enrollment rates and reasons for non-enrollment were analyzed in 622 eligible children: 165 pre-COVID-19 (November 2017–August 2019) and 457 post-COVID-19 (May 2022–August 2024).

**Main results:**

Enrollment rates declined from 30.9% pre-COVID-19 to 18.2% post-COVID-19 (*P* = 0.001). Reasons for non-enrollment differed significantly (*P* = 0.001). Parental unavailability decreased post-COVID-19 (17.7% vs. 34.2%, *P* < 0.001), while research staff unavailability increased (28.6% vs. 15.8%, *P* = 0.006). Overall parental refusal rates remained similar (38.6% pre-COVID-19 vs. 39.6% post-COVID-19, *P* = 0.85). However, among all eligible patients, enrollment failure due to parental refusal increased post-COVID-19 (64.1% vs. 46.3%, *P* = 0.003). Parental refusal inversely correlated with research staff availability (*r* = −0.71, *P* = 0.003).

**Conclusions:**

The COVID-19 pandemic is associated with lower enrollment rates in RCTs enrolling critically ill children. Increased parental refusal post-pandemic is confounded by reduced research staff availability. Further investigation is needed to assess the role of science denialism and identify strategies to enhance enrollment in RCTs of critically ill children.

## Introduction

The Coronavirus Disease 2019 (COVID-19) pandemic profoundly impacted all aspects of healthcare, including clinical research (1). The urgency to gather data on this new health threat led to de-prioritization of non-COVID-19 research. Social distancing mandates, limited access to personal protective equipment, and a shift in focus from research efforts to patient care further decreased enrollment in clinical trials during the height of the pandemic in 2020 (2). The rising prevalence of distrust in science during the pandemic has further complicated enrollment (3). During the initial global spread of the pandemic, 5,758 non-COVID clinical trials were halted, representing a significant increasing trend over time (*p* for trend <0.001) (4). These trends contributed to a nearly 75% reduction in new patient enrollment in non-Covid-19 clinical trials during the pandemic globally (2).

The COVID-19 pandemic exacerbated the already challenging subject enrollment of critically ill children into randomized clinical trials (RCT). There is a critical need for such research, given that many interventions in pediatric critical care are implemented without supporting scientific evidence and are influenced by patient-specific factors, institutional resources, and physician practice styles (5). These RCTs often have narrow enrollment windows, i.e., when the child is critically ill, during a very stressful time for the child and their caregivers (6, 7). Lim et al. showed that caregivers' willingness to participate in clinical research during COVID-19 was impacted by the type of research. In their survey of over 600 caregivers during the pandemic, caregivers of children were much less willing to enroll their children in vaccine-focused trials (22%) compared with non-vaccine research modalities (59%–64%), highlighting how pandemic-specific trial type markedly affects parental decision-making and research hesitancy (8). Furthermore, although children overall experienced lower rates of severe illness from COVID-19, data from a systematic review indicate that previously healthy children still faced an approximate 4% risk of critical disease, while those with one or more comorbidities had substantially elevated odds of critical illness (9). In addition, multinational ICU-based cohorts reported mortality rates in the 20% range for children with critical COVID-19 or MIS-C (10). To date, no research has examined the impact of COVID-19 on enrollment of critically ill children in RCTs pre- and post-pandemic. We had the opportunity to address this question from our 2 similarly designed multicenter RCTs conducted pre- and post-pandemic. We conducted a pilot RCT of pharmacologic prophylaxis against central venous catheter (CVC)-associated deep vein thrombosis (CADVT) in critically ill children pre-pandemic (2017–2019) (11). A subsequent phase 2/3 RCT was conducted post-pandemic (2022–2024) (12). We aimed to evaluate the association of the COVID-19 pandemic with enrollment in RCTs of critically ill children. We hypothesized that enrollment rates declined due to increased parental refusal.

## Materials and methods

This cross-sectional study was conducted to analyze eligible children from both RCTs up to August 2024. Both RCTs were approved by the investigational review board and registered in clinicaltrials.gov (NCT03003390 and NCT04924322).

We previously conducted the Catheter-Related Early Thromboprophylaxis with Enoxaparin (*CRETE*) *Trial* (Appendix A) from November 2017 to August 2019 (pre-COVID-19 RCT). In this phase 2b RCT conducted at 5 centers in the United States, critically ill children <18 years old with a newly inserted CVC were enrolled and randomized to enoxaparin prophylaxis or usual care (11). Serial blood samplings were done for biomarker analyses and ultrasound was performed primarily when the CVC was removed to detect CADVT. Based on findings from the *CRETE Trial*, we designed and subsequently began enrollment in the *CRETE Studies* (Appendix B) beginning in May 2022 (post-COVID-19 RCT) from at least 15 centers in the United States, including the 5 centers in the original *CRETE Trial* (12). The *CRETE Studies* is a phase 2/3 RCT with eligibility criteria identical to the *CRETE Trial*. The study designs differed only in the dose of enoxaparin administered to infants <1 year old randomized to the intervention arm. The *CRETE Trial* demonstrated an apparent difference in treatment effect between older children and infants prompting stratification by age for the *CRETE Studies* and subsequent trials (11). We included all eligible children from the pre-COVID-19 RCT and up to August 2024 from the post-COVID-19 RCT. Enrollment rates and reasons for failure to enroll eligible subjects were tracked in both RCTs. Reasons for failure to enroll were collected and recorded by research staff within a centralized database at times of informed consent. They included parents refused, parents not available, research staff not available, attending declined, ward of the state, and language barriers.

Enrollment rate was defined as the proportion of eligible children who were enrolled in the RCT. Data are presented as counts (percentages) and between group comparisons were performed using chi-squared tests. The Mantel–Haenszel method was used to calculate the pooled odds ratio (OR) and 95% confidence interval (CI) of enrollment rates for the 5 sites that participated in both RCTs. In *post hoc* analysis, adjusted Pearson residuals were calculated to analyze the difference between observed and expected enrollment rates. We also used Pearson correlation coefficient to assess for correlation. All statistical analyses were conducted using StataNow (Version 19.5, StataCorp, College Station, TX). A 2-sided *P* value < 0.05 was considered statistically significant. Adjusted Pearson residuals are normally distributed such that absolute values above 2 indicate statistical significance.

## Results

Of 622 eligible children, 165 (26.5%) were eligible for enrollment in the pre-COVID-19 RCT and 457 (73.5%) were eligible for enrollment in the post-COVID-19 RCT. A total of 51 and 83 children were enrolled in the pre-COVID-19 and post-COVID-19 RCTs, respectively. Enrollment rate was higher in the pre-COVID-19 RCT than in the post-COVID-19 RCT at 30.9% vs. 18.2% (*P* = 0.001). Enrollment rates in the 5 centers that participated in both RCTs ranged from 19.7%–58.8% pre-COVID-19 and 5.3%–46.1% post-COVID-19 for a pooled OR of 0.32 (95% CI: 0.17, 0.61).

**Figure 1:**
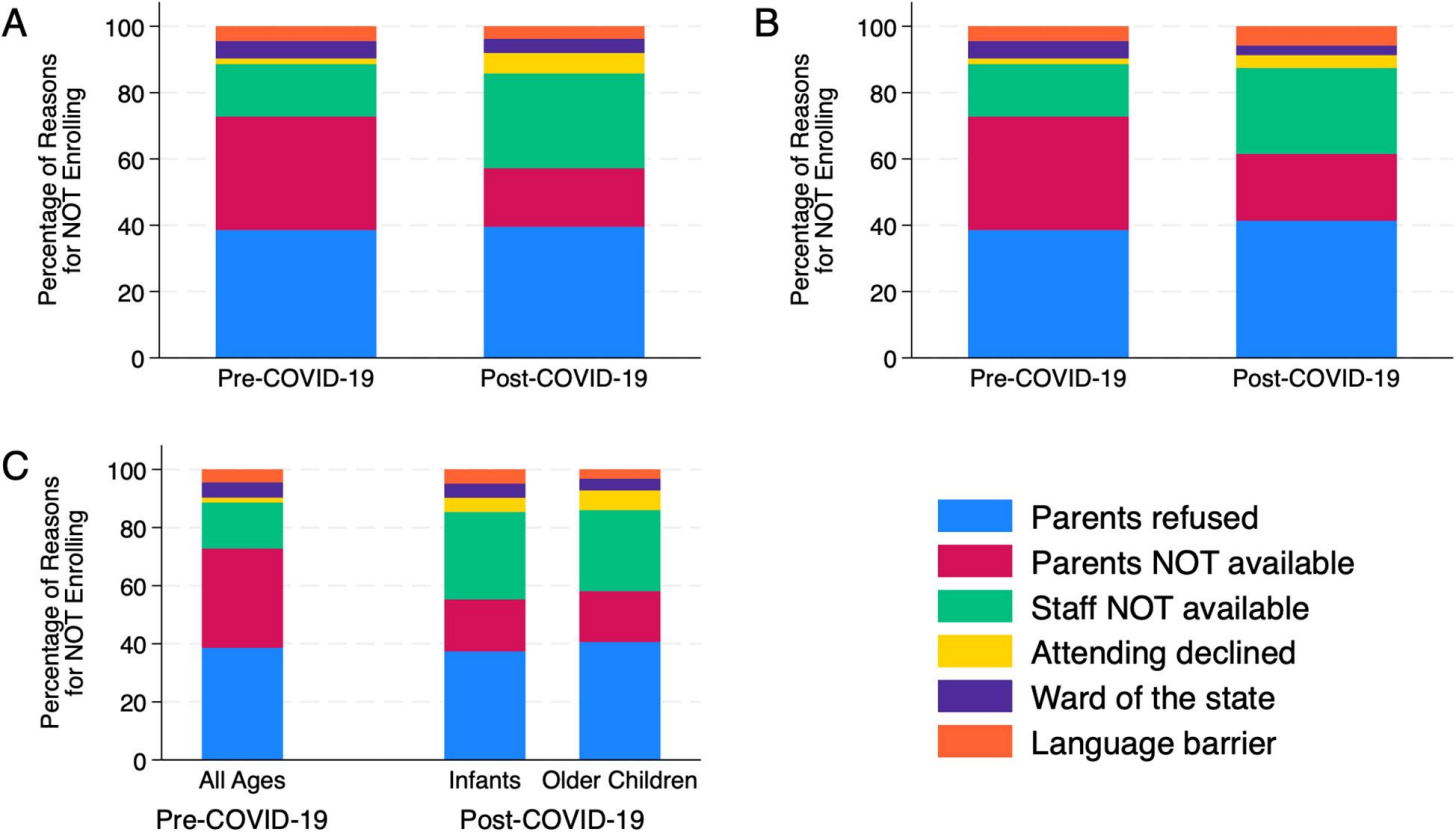
**(A)** Reasons for non-enrollment pre-COVID-19 and post-COVID-19. **(B)** Reasons for non-enrollment pre-COVID-19 and post-COVID-19 across 5 centers who participated in both randomized clinical trials. **(C)** Rate of parent refusal per parents approached.

Among all eligible children, the distribution of reasons for not enrolling differed between the pre- and post-COVID-19 RCTs (*P* = 0.001) (Figure 1A). Parental unavailability was proportionally lower in the post-COVID-19 RCT (17.7% vs. 34.2% pre-COVID-19 RCT, adjusted residual: −3.8). In contrast, the unavailability of research staff was proportionally higher in the post-COVID-19 RCT (28.6% vs. 15.8% pre-COVID-19 RCT, adjusted residual: −2.7). Parental refusal for all eligible children was comparable in the pre- and post-COVID-19 RCTs (38.6% vs. 39.6%, adjusted residual: 0.2). For the 5 centers that participated in both RCTs, a comparable breakdown was seen (*P* = 0.13) (Figure 1B). In the post-COVID-19 RCT, which stratified enrollment between infants (*n* = 140) and older children (*n* = 317), the distribution of reasons for not enrolling were comparable between age groups (*P* = 0.90) (Figure 1C).

**Figure 2:**
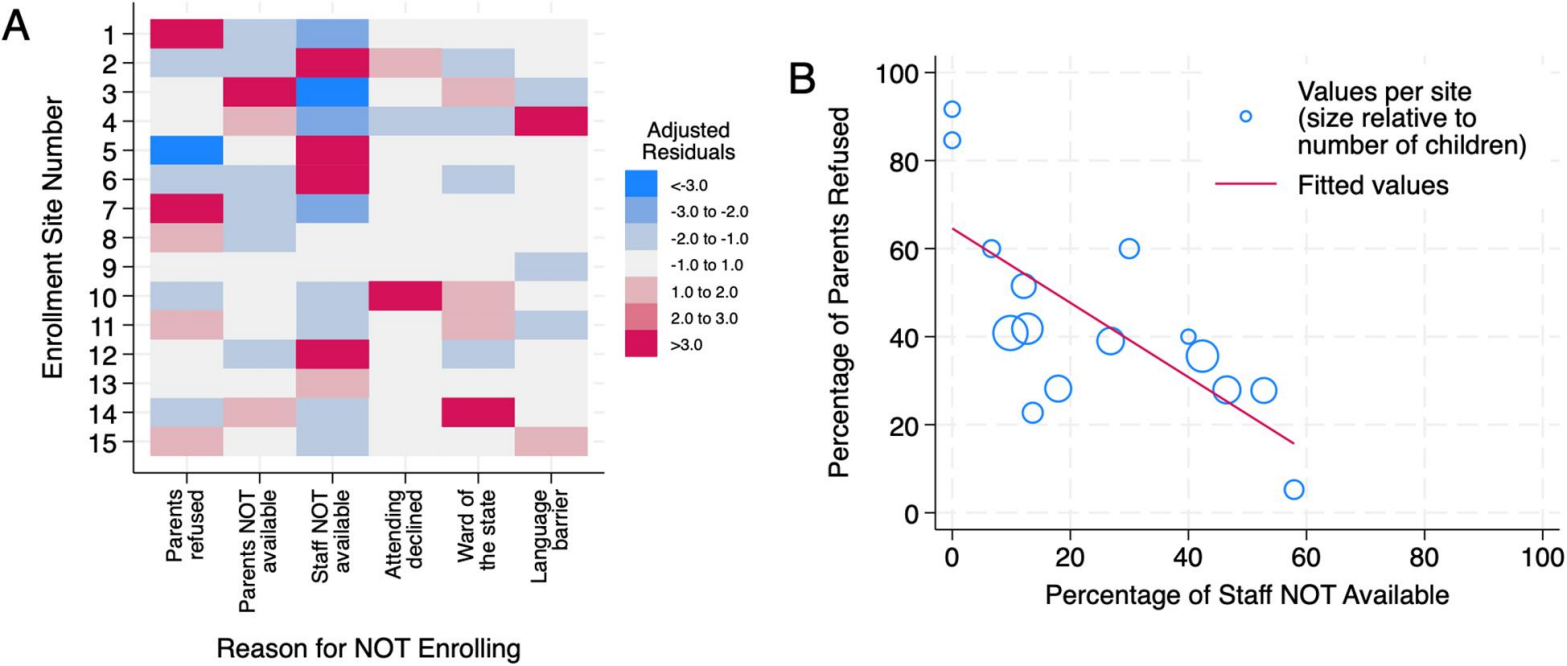
**(A)** Reasons for not enrolling per center. **(B)** Parental refusal per hospital inversely correlated with research staff availability.

We further analyzed the reasons for not enrolling eligible children within each center (Figure 2A). The percentage of parental refusal per site was inversely correlated with the percentage of unavailability of research staff (correlation coefficient: −0.71, *P* = 0.003) (Figure 2B). When considering only children whose parents were approached for enrollment, the percentage of parental refusal was higher in the post-COVID-19 RCT (*n* = 231) than in the pre-COVID-19 RCT (*n* = 95), 64.1% vs. 46.3% (*P* = 0.003).

## Discussion

In this analysis of 2 comparable RCTs of critically ill children conducted pre- and post-COVID-19, we found lower enrollment rates post-COVID-19. Proportionally, parental unavailability was lower, while unavailability of research staff was higher, post-COVID-19. Consistent with our hypothesis, parental refusal was proportionally higher post-COVID-19 when considering only those children whose parents were approached for enrollment. We also did not find a significant difference in the distribution of reasons for not enrolling when stratified by age.

The reasons underlying higher parental refusal post-COVID-19 in our study are unclear, likely multifactorial and would need in-depth qualitative studies. Age of the child is one factor to explore in parents' willingness to consent. Previously, Nasef et al. found that children whose parents consented to research were older than those who declined (13). This was contrary to our findings. Additionally, the trial design itself may affect parental consent rates. Among drug trials in neonates, Hanvey et al. noted that shorter studies <24 h had higher rates of consent (14). Finally, in a systematic review of barriers to enrollment in pediatric clinical research studies, the authors identified trust in medical professionals as a key factor influencing parental participation (15). Moreover, science denialism, defined as the rejection or distortion of established scientific evidence, often driven by ideological, political, or financial motives, has become pervasive following the COVID-19 pandemic and may partly explain our findings (16). Science denialism has likely heightened parental concerns regarding medical interventions and clinical research. Addressing science denialism through targeted education and community engagement is essential to rebuilding trust and may improve participation in pediatric RCTs. We found higher rates of parent refusal post-COVID-19 only among those who were approached for consent. It is unknown whether factors such as trust in medical professionals or science denialism played a role in decision making for these parents.

Our work highlights additional challenges for enrollment, given potentially fewer opportunities for research staff to engage families. The COVID-19 pandemic has led to a boom in the work-from-home or hybrid arrangements. Like our study, Armstrong and colleagues recently reported that 35% of children eligible for studies in their intensive care units did not have any parents available to consent (17). They did not report on parental availability relative to the COVID-19 pandemic. While our study showed that parents were more available post-COVID-19, the research staff was not as they may also be mostly working from home. It is likely that reduced research staff availability hindered parental engagement, research promotion, and trust with the medical teams, potentially contributing to lower enrollment rates. Presence of research staff is particularly essential in enrolling critically ill children as direct in person interactions help build trust and provide necessary reassurance to combat the growing skepticism towards research (18). Alternative models of consent that account for in person unavailability for research staff or parents, such as remote consent, opt-out, or deferred consent should be strongly considered (19, 20). These alternative consent models should be considered when planning trials in pediatric critical care, especially for those that involve narrow enrollment windows.

Clear, standard definitions are needed to better understand the implications of these results and to ensure comparability of results across studies. Enrollment rates in studies of critically ill children range from 42%–94% (21). However, enrollment rates are defined differently across studies. As in our study, some define “enrollment rate” as proportion of total number of eligible children, while others define it as a proportion of only eligible children whose parents were approached for consent (22). We suggest that “consent rate” be used to define the proportion enrolled among eligible children whose parents were approached for consent. Depending on the intent, either or both rates should be reported. In our study, we would have missed the higher proportions of parental refusal if we only analyzed the enrollment rate.

Our study has limitations. First, because our study focused on RCTs evaluating prophylaxis in critically ill children, the effects of the COVID-19 pandemic may not be generalizable to observational or therapeutic studies or to non-critically ill populations. Moreover, our design precludes causality. While we specifically asked parents about reasons for refusal, it is possible that other factors contributed to their decision. We did not collect data on race, ethnicity, language, or socioeconomic background for children who were not enrolled in the RCTs. These are relevant factors known to influence parental decision-making and may impact enrollment patterns across centers and time periods. It is also possible that secular trends including policy changes or evolving societal attitudes independent of the pandemic may have impacted trial enrollment. Center-level differences in staffing structures and research culture may have contributed to the differences in enrollment. The reasons underlying higher parental refusal post-COVID-19 in our study are unclear, but likely multifactorial and warrant further qualitative studies to understand fully.

## Conclusions

The COVID-19 pandemic is associated with lower enrollment rates of critically ill children in RCTs, in part, due to increased parental refusal. Further studies should explore the impact of science denialism in parental refusal to identify interventions that may increase enrollment of critically ill children in RCTs. Interventions to address the impact of unavailability of research staff should also be addressed.

## Data Availability

The data analyzed in this study is subject to the following licenses/restrictions: Ongoing clinical trial so unable to release data. Requests to access these datasets should be directed to sarah.kandil@yale.edu.

## Appendix

### A CRETE trial investigators

Clinical Coordinating Center: E. Vincent S. Faustino, Philip Spinella, Leslie Raffini, Sarah Kandil, Tara McPartland, Asaad Awan, Amy Hummel, Matthew Duplin, Oluwanisola Odesina; Data Coordinating Center: Veronika Shabanova, Marilyn Stolar, Xin Hu, I-Hsin Lin; Outcomes Adjudication Committee: Cicero T. Silva, Monica Epelman, Oscar M. Navarro; Data and Safety Monitoring Board: Ranjit Chima, Brian Branchford, Theresa Weiss, Daniel Zelterman; Independent Safety Monitor: Anjali Sharathkumar, Lee Polikoff; Children's Hospital Wisconsin: Sheila Hanson, Tom Nelson, Matthew Plunk, Sadaf Shad, Katherine Siegel; Dell Children's Medical Center: Renee Higgerson, LeeAnn Christie, Saurabh Guleria, Eimeira Padilla-Tolentino, Carolyn E. Ragsdale; Maria Fareri Children's Hospital: Simon Li, Matthew Pinto, Gita Blitshteyn, Adele R. Brudnicki, William Cuddy, Malgorzata Michalowska-Suterska; St. Louis Children's Hospital: Philip Spinella, Manju Abrahm, Tina Barrale, Maraya Camazine, Juliana DaFonseca, Meghan Huff, Lana Mehanovic, Jennifer Nicholas, Brad Rider, Sharon Rogers, Stephanie Schafer, Kimberly A. Thomas; University of Rochester Golisano Children's Hospital: Jill M. Cholette, Stephen A. Bean, Mitchell Chess, Carole Cole, Eileen Taillie; Weill Cornell Medical Center: Marianne E. Nellis, Arzu Kovanlikaya, Antonio Rivera-Lopez, Keshia O. Small; Yale-New Haven Children's Hospital: E. Vincent S. Faustino, Anjali Gupta, Nancy Hayes, Sarah Kandil, Joana Rhieu, Cicero T. Silva, Joana Tala.

### B CRETE studies investigators

Clinical Coordinating Center: E. Vincent S. Faustino, Laura Holtz, Tara McPartland, Zi Kai Soo; Executive Committee: Sarah Kandil, Leslie Raffini, Cicero Silva, Philip Spinella, Sarah Taylor; Data Coordinating Center: Veronika Shabanova, Sarah McCullom; Outcomes Committee: Cicero T. Silva, Lauren Ehrlich, Oscar M. Navarro; Data and Safety Monitoring Board: Ryan Barbaro, Julie Jaffray, Joshua Warren; Independent Safety Monitor: Anjali Sharathkumar, Lee Polikoff; Children's Hospital Colorado: Matthew K. Leroue, Venessa Hoppes Arellano, Alix Andreaco, Natasha Baig, Ellen Burke, Todd Carpenter, Lanae Dailey, Allie Elozory, Obehi Enabulele, Christiane Furleng, Kristen Gonzales, Katie Gordon, Sharon Gordon, Rachel Greer, Allison Grimsley, Rachel Jones, Michelle Landis, Jaime Laurin, Emily Lipani, Aline Maddux, Rachel Mansour, Mya Merrow, Lexi Patruccelli, Emily Port, Heidi Sauceda, Elizabeth Shields, Yamila Sierra, Erin Stenson, Christine Stewart, Jeffrey Tutman, Patrick Wilson, Ariana Valenzuela, Frances Zorensky; Children's Hospital of Philadelphia: Christie L. Glau, Teresa Arroyo, Ryan Burnett, Jenny Bush, Shawn Dickey, Rebecca Douglas, Glory Edioma, Stephen Famularo, Julie Fitzgerald, Adam Himebauch, Nola Juste, Cindee Levow, Alanah McKelvey, Myooran Sivarupan, Taylor Slocumb, Wandave Tizhe, Katie Tolkacheva, Krithika Tupil; Children's Hospital of Richmond: Vu Nguyen, Megan Scott; Children's Hospital St. Louis: Amanda Kolmar, Jessie Archie-Dilworth, Michael Kramer, Pamela Stone; Children's Hospital Wisconsin: Hilary Schreiber, Miranda Privatt, Sadaf Shad; Children's of Alabama: Michele Kong, Candice Colston, Heather Kelley, Meghan Murdock; Golisano Children's Hospital: Jill M. Cholette, Eileen Root Taillie; Hassenfeld Children's Hospital: Michelle Ramirez, Sandra Deygoo, Kelley Groves, Claire Hennigan, Jaclyn McKinstry, Michael Martinez, Rosa Michos, Ami Shah, Maria Spilios; Johns Hopkins All Children's Hospital: Anthony A. Sochet, Tamara Babushkin, Hanna Cree, Lexie Dallas, I. Federico F. Nievas, Beatriz Teppa; Maria Fareri Children's Hospital: Matthew G. Pinto, Sarah Korn, Julie Levasseur, Serè Politano; Nationwide Children's Hospital: Jennifer Muszynski, Lisa Steele; New York Presbyterian Hospital: Marianne E. Nellis, Liliko Chung, Margaret Cormack, Oleksiy Svezhenets; Oklahoma Children's Hospital: Teddy M. Muisyo, Christine Allen, Ashley Anderson, Karl M. Huebner, Tracy Jones, Katherine Mitchell, Kaci Taylor; Penn State Hershey Children's Hospital: Elizabeth Kerris, Hannah Hamilton, Debra Spears, Neal Thomas; Stead Family Children's Hospital: Madhuradhar Chegondi, Maureen Austin, Mahil Rao; Yale-New Haven Children's Hospital: E. Vincent S. Faustino, Shelby Blalock, Michelle Ecarma, Nancy Hayes, Sarah Kandil, Matthew Kluko, Tyler Quinn, Cicero T. Silva.
